# Histone Deacetylases and Their Inhibition in *Candida* Species

**DOI:** 10.3389/fmicb.2016.01238

**Published:** 2016-08-05

**Authors:** Cécile Garnaud, Morgane Champleboux, Danièle Maubon, Muriel Cornet, Jérôme Govin

**Affiliations:** ^1^Laboratoire de Parasitologie-Mycologie, Institut de Biologie et de Pathologie, Centre Hospitalier Universitaire Grenoble AlpesGrenoble, France; ^2^Laboratoire TIMC-IMAG-TheREx, UMR 5525 CNRS-UGA, Université Grenoble AlpesGrenoble, France; ^3^Université Grenoble Alpes, Institut National de la Santé et de la Recherche Médicale U1038, Commissariat à l’Énergie Atomique et aux Énergies Alternatives, Biosciences and Biotechnology Institute of Grenoble – Large Scale Biology LaboratoryGrenoble, France

**Keywords:** HDAC, chromatin, acetylation, *Candida*, HDAC inhibitors

## Abstract

Fungi are generally benign members of the human mucosal flora or live as saprophytes in the environment. However, they can become pathogenic, leading to invasive and life threatening infections in vulnerable patients. These invasive fungal infections are regarded as a major public health problem on a similar scale to tuberculosis or malaria. Current treatment for these infections is based on only four available drug classes. This limited therapeutic arsenal and the emergence of drug-resistant strains are a matter of concern due to the growing number of patients to be treated, and new therapeutic strategies are urgently needed. Adaptation of fungi to drug pressure involves transcriptional regulation, in which chromatin dynamics and histone modifications play a major role. Histone deacetylases (HDACs) remove acetyl groups from histones and actively participate in controlling stress responses. HDAC inhibition has been shown to limit fungal development, virulence, biofilm formation, and dissemination in the infected host, while also improving the efficacy of existing antifungal drugs toward *Candida* spp. In this article, we review the functional roles of HDACs and the biological effects of HDAC inhibitors on *Candida* spp., highlighting the correlations between their pathogenic effects *in vitro* and *in vivo*. We focus on how HDAC inhibitors could be used to treat invasive candidiasis while also reviewing recent developments in their clinical evaluation.

Invasive fungal infections have become a major public health problem, with up to two million cases worldwide each year (Brown et al., 2012). In developed countries, disseminated candidiasis, mostly caused by the yeasts *Candida albicans, C. glabrata*, and *C. parapsilosis*, remains the predominant threat, with more than 400,000 cases per year (Brown et al., 2012). Antifungal treatments are currently based on only four classes of drugs: polyenes, principally represented by amphotericin B; triazoles; echinocandins; and pyrimidines (Denning and Bromley, 2015). The emergence of strains resistant to this limited arsenal makes the need for novel therapeutic agents urgent (Denning and Bromley, 2015).

*Candida albicans* is the predominant cause of invasive candidiasis, and is also the most extensively studied *Candida* species. Its great success as a pathogen is linked to its capacity to survive in the bloodstream, to invade tissues and to effectively adapt to a range of host niches. One of its key virulence traits is its morphological plasticity; its ability to shift from a yeast form to a hyphal form has been clearly linked to virulence (Sudbery, 2011; Gow et al., 2012). Hyphal forms adhere better to mucosal niches, making it easier to maintain their colonization. This colonization can lead to epithelial rupture, dissemination of the pathogen in the bloodstream and ultimately invasion of deep-seated tissues. Other virulence factors, such as the white-to-opaque switch, the GUT (Gastrointestinally IndUced Transition) or gray phenotypes, cell wall plasticity, adherence, and biofilm formation favor development in the host (Polke et al., 2015).

*Candida albicans*’ capacity to adapt to various environmental conditions, including drug pressure, is linked to a complex interplay of stress-signaling responses (Fuchs and Mylonakis, 2009; Shor and Perlin, 2015). This signaling alters the transcription program to adapt the production of proteins, causing the emergence of the biological state that will be the most beneficial for yeast survival and development. Transcriptional regulation requires transcription factors to bind their DNA template and subsequently recruit dedicated machinery for transcription repression or activation. Classical histone modifications, such as acetylation, methylation, and phosphorylation have been shown to play a role in regulating stress responses, antifungal tolerance and virulence in *C. albicans* and *C. glabrata* (Liu et al., 2005; Rai et al., 2012; Stevenson and Liu, 2013; Kim et al., 2015; Tscherner et al., 2015). In particular, reversible acetylation by various histone acetyltransferases (HATs) and histone deacetylases (HDACs, also known as lysine deacetylases or KDACs) is crucial to chromatin-mediated transcriptional regulation. Recent studies have suggested that inhibiting fungal HDACs may have beneficial and synergistic effects, reducing the virulence and growth of *Candida* spp., while also decreasing their tolerance and resistance to existing antifungal drugs (Al-Dhaheri and Douglas, 2010; Wurtele et al., 2010; Stevenson and Liu, 2011; Hnisz et al., 2012; Lu et al., 2012; Nobile et al., 2014; Rajasekharan et al., 2015; Li et al., 2015; Pfaller et al., 2015; Zhang and Xu, 2015).

In this review, we briefly summarize the advances made in the characterization of HDACs in *Candida* spp. We have also correlated the functional roles of HDACs and the *in vitro* biological properties of HDAC inhibitors on *Candida* spp., mostly *C. albicans*, with their *in vivo* effects and discussed the potential for development of new antifungal compounds.

## HDACs In *Candida* Species

**FIGURE 1:**
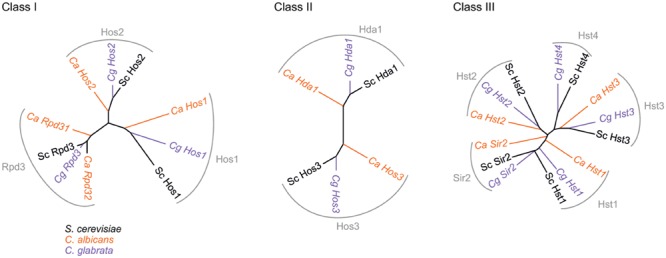
**Phylogenetic trees representing HDACs in *S. cerevisiae, C. albicans*, and *C. glabrata*.** Percent identities matrices between these HDACs are presented in Supplementary Table S1.

To date, a total of 11 HDACs have been identified in *C. albicans* and *C. glabrata* (**Figure 1**; **Tables 1** and **2**). These two species represent the major proportion of the fungal infections clinically observed and regroup most of the molecular information accumulated on fungal HDACs. Three main classes of HDACs have been originally described in *C. albicans* based on the key *S. cerevisiae* enzymes (Trojer et al., 2003; Kim et al., 2015). The key enzyme for Class I HDACs is Rpd3, for Class II it is Hda1, and for Class III it is Sir2, a sirtuin. This family of enzymes uses a specific enzymatic chemistry based on the cofactor nicotinamide adenine dinucleotide (NAD).

**Table 1:** Gene and protein accession numbers for HDACs expressed in *C. albicans*.

Classes		Name	Gene name	Protein ID	Reference
I	Rpd3 type	Rpd31	CR_02760C	Q5A209	Srikantha et al., 2001
		Rpd32	C3_07000W	Q5ADP0	Hnisz et al., 2009
		Hos1	C4_06010C	Q59Q78	Srikantha et al., 2001
		Hos2	C3_00780W	Q5A839 Q5A7T9	Srikantha et al., 2001
II	Hda1 type	Hda1	CR_02050C	Q5A960	Klar et al., 2001; Srikantha et al., 2001; Hnisz et al., 2009; Zacchi et al., 2010
		Hos3	C4_02300W	Q5AF34	Srikantha et al., 2001
III	Sirtuin	Sir2	C2_01330C	O59923	Klar et al., 2001; Maglott et al., 2007; Fu et al., 2008; Nobile et al., 2012
		Hst1	C1_09050W	Q5AQ47	Maglott et al., 2007; Hnisz et al., 2009
		Hst2	CR_01800C	Q5A985	Maglott et al., 2007; Hnisz et al., 2009; Nobile et al., 2012
		Hst3	C5_01340W	Q5A1W9	Enjalbert et al., 2006; Maglott et al., 2007; Wurtele et al., 2010; Singh et al., 2011; Stevenson and Liu, 2011
	Fungi only	Set3	C1_14140C_A	Q59ZX1	Uhl et al., 2003; Maglott et al., 2007; Hnisz et al., 2009, 2010

**Table 2:** Gene and protein accession numbers for HDACs expressed in *C. glabrata*.

Classes		Name	Gene name	Protein ID	Reference
I	Rpd3 type	Rpd3	CAGL0B01441g	Q6FXA7	This study
		Hos1	CAGL0D01430g	Q6FWB7	Dujon et al., 2004
		Hos2	CAGL0A03322g	Q6FY81	This study
II	Hda1 type	Hda1	CAGL0J03454g	Q6FPJ0	This study
		Hos3	CAGL0J06974g	Q6FP35	Dujon et al., 2004
III	Sirtuin	Sir2	CAGL0C05357g	Q6FWI7	Dujon et al., 2004
		Hst1	CAGL0K01463g	Q6FNA6	This study
		Hst2	CAGL0L08668g	Q6FKU1	Dujon et al., 2004; Domergue et al., 2005
		Hst3	CAGL0H08239g	Q6FRI7	Dujon et al., 2004
		Hst4	CAGL0F05621g	Q6FU79	This study
	Fungi only	Set3	CAGL0G04499g	Q6FT89	This study

### Class I HDACs: Rpd31, Rpd32, Hos1, and Hos2

Interestingly, *C. albicans* possesses two genes which are potential orthologs of *S. cerevisiae*’s *RPD3*. These genes are now designated as *RPD31* and *RPD32*. The current annotation of these genes has given rise to some confusion, with discrepancies between the original publication (Srikantha et al., 2001) and the current annotation in the Candida Genome Database (CGD; Assembly 22, version s06-m01-r01). **Table 1** presents updated information on the gene and protein accession numbers. Hos1 was first identified and cloned 15 years ago in *C. albicans*, but its functional role has remained elusive (Srikantha et al., 2001). Hos2 was initially described in the CGD as a Class III enzyme with sirtuin activity (Karthikeyan et al., 2013), but it is now presented as a member of the class I family (Kim et al., 2015). *In vitro* analysis of the enzymatic activity of recombinant Hos2 showed it to be inactive on acetylated histones but capable of deacetylating acetylated tubulin (Karthikeyan et al., 2013). These findings remain to be confirmed *in vivo*.

### Class II HDACs: Hda1 and Hos3

Hda1 was identified in 2001 and was shown to play an important role in hyphal development (see below). Hos3 was also described in 2001, but its function has yet to be studied in detail in *Candida* spp. (Srikantha et al., 2001).

### Class III HDACs: Sir2 and Hst Proteins

The sirtuin family, a group of NAD+-dependent HDACs, is conserved between some yeasts and humans. Sirtuins were first characterized in *Candida* spp. in 1999, with the identification and cloning of the *SIR2* gene in *C. albicans* (Pérez-Martín et al., 1999). Sir2 deacetylates histones, specifically lysine 16 on histone H4, it is also important for silencing at telomeres and ribosomal genomic regions (Freire-Benéitez et al., 2016). Interestingly, *SIR2* is not present in all *Candida* spp. Thus, for example in *C. lusitaniae*, no Sir functionality for heterochromatic silencing in subtelomeric and pericentric regions has been detected, while in other species such as *C. albicans* or *C. glabrata*, an ancestral gene was duplicated to generate *HST1* and *SIR2* (Froyd et al., 2013; Kapoor et al., 2015).

Hst are also members of the sirtuin family; Hst1 is a component of the Set3 HDAC complex, while Hst3 is involved in nucleosome assembly. With the HAT Rtt109, Hst3 dynamically controls the level of lysine 56 acetylation on histone H3 (Rundlett et al., 1996; Wurtele et al., 2010).

### Other HDACs: Set3

Set3 is an NAD+-dependent HDAC, which, in *S. cerevisiae*, forms a 7-subunit complex (Set3C) containing HDAC and non-HDAC proteins in *C. albicans* (Hnisz et al., 2010, p. 2). Four of these proteins, Set3, Hos2, Snt1 and Sif2, constitute the core complex and are essential for Set3C assembly, while three others (Hos4, Hst1, and Cpr1) are peripheral. Set3, Hos2, and Hst1 have HDAC activity. In addition, the PhD finger domain of Set3 binds methylated H3K4 and recruits the Set3C complex to chromatin in *S. cerevisiae* (Kim et al., 2012). This complex is conserved in *C. albicans*, where it is important for morphogenesis (Nobile et al., 2014).

## Functional Roles of HDACs in *Candida albicans*

### HDACs and Yeast-to-Hyphae Transition

*Candida albicans* exists in various morphological forms: an ovoid-shaped yeast phase is commonly found on mucosal and skin surfaces, where it is well tolerated by the immune system; hyphal forms possess a long tube-like extension to provide increased potential invasiveness. Both forms contribute to disseminated infections, but the ability to reversibly switch from one form to the other has been directly linked to virulence. The yeast-to-hyphae transition is controlled by various pathways which were recently reviewed (Sudbery, 2011).

**FIGURE 2:**
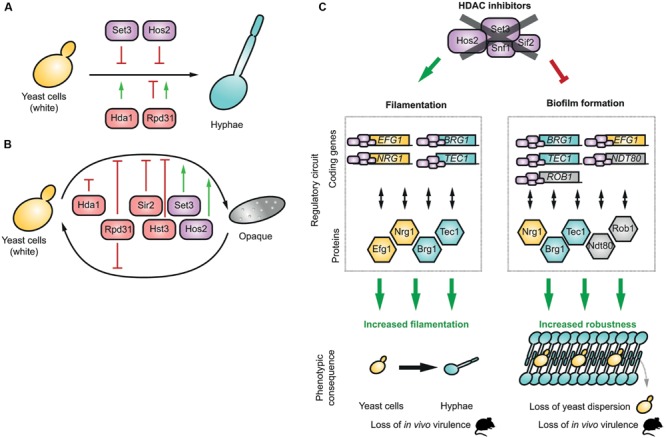
**HDACs and morphogenesis in *C. albicans.*** Phenotypic effect of different HDACs during filamentation **(A)** and the white to opaque transition **(B), (C)** HDACs regulate the expression of key transcription factors of regulatory circuits, which regulate the gene expression program during filamentation and the formation of biofilms. HDAC inhibition deregulates the transcription regulatory circuit, generating a hyperfilamentation phenotype and a loss of virulence *in vivo*. Similarly, upon treatment with HDAC inhibitors, biofilms are more robust but have a decreased yeast dispersion and a loss of virulence *in vivo*.

The functional role of many HDACs has been linked to the yeast-to-hyphae transition (**Figure 2A**). First, Hda1 was reported to be important for a specific chromatin state during hyphal elongation and maintenance (Lu et al., 2011); *C. albicans* strains deleted for the *HDA1* gene are unable to maintain hyphal development. Hda1 is recruited by the transcription factor Brg1 and establishes a chromatin state which is not permissive to Nrg1 repressor binding. Thus, Nrg1 is unable to bind the promoter regions of hypha-specific genes and prevent their expression (Lu et al., 2011, 2012). And in the absence of Nrg1, Hda1 also maintains a nucleosomal structure compatible with the expression of the hyphal genes.

The yeast-to-hyphae transition is also controlled by Rpd31, which acts both as a repressor and an activator (**Figure 2A**). In yeast cells, Rpd31 is repressing the expression of hyphal-specific genes such as *HWP1* and *ECE1* and under non-hyphae-inducing conditions, these genes are activated when *RPD31* or *SSN6* are deleted (Lee et al., 2015). However, under filament-inducing conditions, the Rpd31-Ssn6 complex promotes filamentous elongation by triggering the expression of the master regulator *UME6*, a key factor in hyphal differentiation (Lee et al., 2015). Finally, the Set3C complex has been identified as a repressor of the yeast-to-hyphae transition (**Figure 2A**). In hyphae-inducing conditions *in vitro, set3-*, and *hos2-*null mutant strains had hyperfilamentous phenotypes (Hnisz et al., 2010, p. 3). The same authors later showed that the *set3* mutant induces transient upregulation of *EFG1* and *NRG1*, and downregulation of other hyphal associated genes, such as *BRG1* and *TEC1* (Hnisz et al., 2012).

### HDACs and the White-to-Opaque Transition

*Candida albicans* colonies are typically white and smooth, but under some specific conditions, such as genetic conversion at the mating-type locus, a morphological white-to-opaque switch can occur. White cells have been showed to be more virulent in murine models (Kvaal et al., 1997). In human systemic infections, white cells are more likely to be isolated, whereas opaque cells may be better adapted to colonization (Morschhäuser, 2010).

The white-to-opaque transition involves a set of transcription factors responsible for the control of genes specific to white and opaque cells (Hernday et al., 2013). These factors are naturally linked to the transcription and chromatin machinery. Strains deleted for either *HDA1* or *RPD31* showed an enhanced ability to switch from the white to the opaque state, while only the *rpd31Δ* mutant displayed increased reverse opaque-to-white switch (**Figure 2B**). Thus, Hda1 selectively represses the white-to-opaque switch and Rpd31 suppresses the transition in both directions (Klar et al., 2001; Srikantha et al., 2001).

The sirtuins Hst3 and Sir2 were identified as switch repressors, whereas Set3C HDACs (Set3 and Hos2) were recognized as key activators of the white-to-opaque switch (**Figure 1**) (Pérez-Martín et al., 1999; Hnisz et al., 2009; Stevenson and Liu, 2011).

### HDACs and Biofilm Formation

*Candida albicans* can form biofilms – multicellular structures of mixed communities of microorganisms containing yeast and hyphal forms surrounded by a self-produced extracellular matrix – which commonly develop on implanted medical devices, such as intravascular catheters or prostheses, as well as on mucosal surfaces. Biofilms create secondary infectious foci in hematogenous disseminated candidiasis through the release of yeast cells into the bloodstream. They are also an important source of antifungal resistance because the extracellular matrix hinders drug diffusion (Taff et al., 2013; Perlin et al., 2015).

Set3C HDACs have been shown to be important for the development of biofilms (Nobile et al., 2014). Thus, deletion of *SET3* and *HOS2* decreases biofilm formation and biomass, and these mutants seem to be more resistant to mechanical shearing and yeast dispersion *in vivo* (Nobile et al., 2014). The Set3C complex binds to five of the six biofilm master regulators, namely *NRG1, BRG1, TEC1, NDT80*, and *ROB1*. Notably, Nrg1, which is transiently repressed by Set3C during filamentation, is involved in the regulation of cellular dispersion (Uppuluri et al., 2010).

### Role of HDACs in Virulence *in vivo*

Several studies have investigated the role of HDACs in *C. albicans* virulence through *in vivo* experiments, mostly assessing survival rates after systemic injection of wild-type and mutant strains.

The *RPD31* deletion induced filamentation defects and attenuated virulence when injected into mice (Srikantha et al., 2001; Lee et al., 2015). These results are consistent with the hyphae-inducing conditions found in animal models. Likewise, the *set3* mutant displayed a hyperfilamentous phenotype *in vitro.* This phenotype was confirmed *in vivo* in mouse kidneys, but, surprisingly, it was associated with attenuated virulence (Hnisz et al., 2010) (**Figure 2C**). This attenuated virulence could be linked to the Set3C-mediated transcription regulation which includes transient downregulation of *EFG1* and *NRG1* and induction of *BRG1* and *TEC1* (Hnisz et al., 2012). In addition, Hst3 deletion leads to increased H3K56 acetylation, decreased cell viability with abnormal filamentous growth and genomic instability. *In vivo*, this deletion attenuates the virulence of *C. albicans* in mice models (Wurtele et al., 2010).

### HDACs and Antifungal Resistance

Histone acetylation dynamics and HDACs have been shown to be involved in the development of resistance to antifungal drugs. Notably, Li et al. (2015) showed that the expression of *HDA1* and *RPD3* was increased during acquisition of azole resistance, but decreased once resistance had been established. Hda1 and Rpd3 control the acetylation of Hsp90, a protein involved in the development of drug resistance in various fungi (Cowen and Lindquist, 2005; Robbins et al., 2012). Inactivation of HDACs in *C. albicans* phenocopies the genetic and pharmacological inhibition of Hsp90, restoring azole susceptibility by blocking the Hsp90-dependent response involved in azole resistance (Cowen and Lindquist, 2005).

Some authors initially hypothesized that this effect was achieved because HDACs directly influenced the expression of eﬄux transporter genes involved in azole resistance. However, recent studies suggest that the deletion of HDACs or the use of HDAC inhibitors could decrease the expression of eﬄux transporters as a part of a general decrease of histone acetylation and its consequence on transcription regulation (Li et al., 2015).

## Functional Roles of HDACs in *Candida glabrata*

In the species distribution of invasive candidiasis, *C. glabrata* ranks second after *C. albicans* (Brown et al., 2012). Chromatin remodeling and sirtuin-family HDACs are required for *C. glabrata* to adapt to stressful conditions such as survival inside phagocytes, adhesion and maintenance of colonization, or multidrug resistance (Rai et al., 2012; Orta-Zavalza et al., 2013; De Las Peñas et al., 2015).

Sir2 is important for the regulation of cell adhesion; its absence reduces silencing and many subtelomeric adhesin-encoding EPA genes are derepressed (De Las Peñas et al., 2015). Hst1 in *C. glabrata* is recruited by the transcription factor Sum1 and contributes to the repression of *PDR1* and *CDR1*, which regulate the expression of eﬄux pumps. Thus, when Hst1 is deleted, these genes are upregulated and azole resistance is enhanced (Orta-Zavalza et al., 2013).

## Effects of HDAC Inhibitors on *Candida* spp. *in vitro*

Inhibitors of mammalian HDAC enzymes were first developed nearly 35 years ago. As soon as the first molecules were identified, several pioneer studies analyzed their effects on yeast HDACs.

### Non-selective Inhibitors of Class I and II HDACs

Trichostatin A (TSA) is a well-known HDAC inhibitor. It was first isolated from a culture broth of *Streptomyces platensis* and was initially presented as a fungistatic drug inhibiting growth of *Trichophyton* and *Aspergillus* (Tsuji et al., 1976). Rapidly, however, it was shown to act on the differentiation of mammalian cells and to inhibit their HDACs (Yoshida et al., 1987, 1990). Ten years later, TSA was tested on pathogenic yeasts, including *C. albicans* where it induced a dramatic increase in white-to-opaque transition (Klar et al., 2001). This phenotype is fully compatible with Hda1 and/or Rpd31 inhibition (see above, Klar et al., 2001; Srikantha et al., 2001). TSA was also shown to trigger the yeast-to-hyphae conversion of *C. albicans* through inhibition of Set3C HDACs (Hnisz et al., 2010). Finally, the deletion of Hos2, a Set3C subunit, but none of the other HDACs, phenocopies the TSA induced yeast-to-hyphae transition (Hnisz et al., 2010). No *in vitro* assessment of TSA on *Candida* HDACs has been yet reported but TSA is active on purified Rpd3, Hda1 and Hos3 in *S. cerevisiae* (Carmen et al., 1999). Therefore, the phenotypes observed in *Candida* spp. are likely to be mediated by a direct inhibition of the Rpd3, Hda1, and a Hos2 enzyme as a TSA treatment phenocopies the deletion of these HDACs.

Sodium butyrate is another well-known HDAC inhibitor. In 1978, this fatty acid was shown to inhibit mammalian HDACs. In 2002, it was tested in *C. albicans* with other HDAC inhibitors (Candido et al., 1978; Smith and Edlind, 2002). Sodium butyrate alone was shown to have minimal effects on growth, heat sensitivity, and germ tube formation in *C. albicans* (Smith and Edlind, 2002), although some reports suggested that it inhibited growth and biofilm formation in *C. albicans, C. parapsilosis*, and *Cryptococcus neoformans*, while also enhancing the functions of macrophages *in vitro* (Candido et al., 1978). Whether its effect on HDAC enzymes is direct or not remain to be determined.

Some uracil-based compounds have been identified, among which suberoylanilide hydroxamic acid (SAHA), also known as vorinostat. This compound is currently licensed for clinical use for the treatment of cancer. When tested against *C. albicans* strains, it displayed relatively low antifungal activity (Mai et al., 2007), although another study showed that the same concentration could reduce the pathogenicity of C. *albicans* by decreasing its adherence to cultured human cells by 90%, and significantly inhibiting serum-induced germination (Simonetti et al., 2007). Finally, apicidin, a cyclic tetrapeptide, displays limited direct antifungal activity against *C. albicans* (Smith and Edlind, 2002).

### Sirtuin Inhibitors

Nicotinamide is a vitamin and precursor of NAD+ and a well-described inhibitor of Class III HDACs, including Sir2 (Landry et al., 2000; Bitterman et al., 2002; Sanders et al., 2007). It was shown to have broad antifungal activity against several pathogenic *Candida* and *Aspergillus* species. In particular, the addition of nicotinamide to wild-type *C. albicans* cells led to morphological alterations and strong growth inhibition *in vitro*, these effects are thought to be mediated through inhibition of H3K56 deacetylation (Wurtele et al., 2010).

Thus, the *in vitro* effects of these non-selective HDAC inhibitors used alone were only studied in *C. albicans* and results were somewhat conflicting. Further studies including *C. glabrata* will be needed.

### Selective HDAC Inhibitors

In addition to the pan-HDACs inhibitors, such as TSA and SAHA, a fungal-specific Hos2 inhibitor, MGCD290, has been developed (Pfaller et al., 2009). No enzymatic data currently evaluates its effect on purified Hos2, but *in vitro*, this compound alone showed a modest activity against *Candida* spp., with minimum inhibitory concentrations (MICs) ranging from 0.5 to 16 μg/mL, depending on the species. However, in combination with azoles, MGCD290 was active against a broad range of fungi, including molds such as *Aspergillus* spp., and was promoted as the way forward for the development of a new class of clinical drugs.

More generally, the HDAC enzymatic activity is dependent of key residues which have been highly conserved through evolution (Lombardi et al., 2011 and Supplementary Figure S1). Sequence alignments of class I and class II HDACs from *C. albicans* and human reveal that Set3 is the most divergent enzyme, with <20% of identity with human or other *C. albicans* HDACs (Supplementary Figure S1; Supplementary Table S2). The functional study of this enzyme has demonstrated its importance for the biology of *C. albicans* and its virulence *in vivo* (see section Functional Roles of HDACs in *Candida albicans*). Altogether, Set3 appears to be an exceptional candidate for the development fungal specific HDAC inhibitors. Alternatively, Hos1, Hos3, and Hst3 could also constitute new potential targets (Supplementary Table S2).

A new generation of inhibitors has currently been developed and targets selectively human HDAC isoforms, such as HDAC1/2, HDAC3, or HDAC8 (for review, Falkenberg and Johnstone, 2014). This illustrates that high levels of selectivity can be reached among human HDACs. Similarly, it is very likely that specific compounds could probably target specifically fungal HDACs. Structural studies showed that HDAC8 active site is very malleable and adapts its conformation when different inhibitors are bound (Somoza et al., 2004). Future work will hopefully generate more structural information on fungal HDACs and provide additional insights to the quest for specific fungal inhibitors.

## The Clinical Potential of HDAC Inhibition in *Candida* Infections

### HDAC Inhibitors Bolster Existing Antifungal Drugs and Limit the Emergence of Resistance

When used alone, HDAC inhibitors seem to display only a modest anti-*Candida* activity, however, their potential increases exponentially when they are used in combination with existing antifungal agents. This activity is observed not only with planktonic cells but also with biofilms.

Thus, HDAC inhibitors can significantly enhance azole activity *in vitro* (Smith and Edlind, 2002; Mai et al., 2007; Li et al., 2015). Combination of TSA with fluconazole, itraconazole, or voriconazole significantly reduced trailing growth (the phenotypic expression of drug-tolerance) and/or the azole MICs, in *C. albicans, C. parapsilosis*, and *C. tropicalis* (Smith and Edlind, 2002; Mai et al., 2007). Similar effects were observed in *C. albicans* when SAHA or other hydroxamate-based inhibitors were used in combination with fluconazole. These observations are consistent with the increased azole susceptibility of the *HDA1* or *RPD3* mutants of *C. albicans* (Mai et al., 2007; Zhang and Xu, 2015). MGCD290 also potentiates the activity of triazoles against *Candida* spp. *in vitro* (Pfaller et al., 2009, 2015). This synergy between HDAC inhibitors and antifungals is not limited to azoles, and TSA was shown to enhance the activity of other antifungal agents acting on membrane synthesis, including terbinafine, although it had no effect on the activities of amphotericin B and 5-fluorocytosine (Smith and Edlind, 2002). MGCD290 was also found to potentiate the echinocandins, although this synergistic effect was less pronounced than the effect with azoles (Pfaller et al., 2015).

Apart from its activity against *Candida* spp., MGCD290 was shown to have a synergistic activity with azoles against *Aspergillus, Rhizopus, Mucor, Fusarium, Scedosporium, Rhodotorula*, and *Trichosporon* genus (Pfaller et al., 2009).

One of the growing threats in the treatment of invasive candidiasis is the emergence of multidrug resistance, including echinocandin resistance, especially in *C. glabrata* (Maubon et al., 2014). Synergy between HDAC inhibitors, azoles (especially fluconazole) and echinocandins was also demonstrated for the treatment of several resistant strains of *C. albicans, C. glabrata*, or *C. krusei* (Pfaller et al., 2009; Li et al., 2015, p. 3). *In vitro*, MGCD290 also decreased the echinocandin-resistance of *C. glabrata, C. albicans*, and *C. krusei* isolates. Moreover, in most azole- or echinocandin-resistant strains, combination with a HDAC inhibitor led to a shift from resistance to greater susceptibility (Pfaller et al., 2015). Similarly, several echinocandin- or azole-resistant *C. albicans* isolates were as sensitive to nicotinamide as susceptible strains upon combined treatment with a HDAC inhibitor (Wurtele et al., 2010).

Through similar studies, several HDAC inhibitors were shown to enhance the action of antifungal drugs against fungi present in biofilms. Thus, Al-Dhaheri and Douglas (2010) showed that, in the presence of amphotericin B, TSA or apicidin, sodium butyrate significantly reduced viability of *Candida* spp. in biofilms. Similarly, sodium valproate, an organic compound used as an anticonvulsive agent which has been shown to be a HDAC inhibitor, used in combination with amphotericin B showed synergistic antifungal activity on biofilms produced by *C. albicans, C. krusei*, and *C. parapsilosis* (Göttlicher et al., 2001; Phiel et al., 2001; Al-Dhaheri and Douglas, 2010). Valproate was the most effective agent against *C. krusei*, while butyrate had the greatest impact on *C. albicans*. Using a biofilm formation assay, butyrate alone showed antifungal activity against *C. albicans, C. parapsilosis*, and *C. neoformans* (Hnisz et al., 2012). A combination therapy based on flavonoids and butyrate also significantly reduced a *C. tropicalis* biofilm (Rajasekharan et al., 2015).

Even though they are preliminary, these results with HDAC inhibitors on biofilms are encouraging. Despite their lack of specificity, HDAC inhibitors may be used at high concentrations in the particular context of lock therapy, which involves the direct application of very high local doses of active drugs to contaminated catheters. In addition, combinatorial strategies against biofilms have recently gained interest for the treatment of *Candida* infections associated with biofilms on devices (Liu et al., 2005). Indeed, the disruption of the membrane or the cell wall by antifungal agents may help promote the uptake of compounds that are active inside cells.

### Use of HDAC Inhibitors in Animal Models and Clinical Studies

The animal experiments described above confirmed that genetic inhibition of HDACs produced strains with attenuated virulence (**Figure 2**). These experiments are important for functional studies, but genetic knock-out models are not entirely predictive of the ability of HDAC inhibitors to cure *Candida* infection *in vivo*.

Indeed, until now, only three HDAC inhibitors have been tested as therapeutic agents in animal models: the sirtuin inhibitor nicotinamide (intraperitoneal injection), valproic acid (intraperitoneal injection) and the Hos2 inhibitor MGCD290 (oral route). Wurtele et al. (2010) demonstrated that nicotinamide, mimics the *in vitro* effects of Hst3 repression, leading to a loss of virulence in mice. This antifungal effect requires the presence of the acetyltransferase RTT109 which acetylates H3K56, suggesting that nicotinamide exerts its therapeutic effect through inhibition of Hst3p-mediated H3K56 deacetylation. Paradoxically, the intra-peritoneal injection of high doses of valproic acid in a disseminated mice model of candidiasis was associated with accelerated (mean time to death: 21.5 days vs. >40 days) and increased mortality (44% vs. 75%, *P* = 0.02; Roger et al., 2011). Similarly, MGCD290 was tested in a murine model of invasive candidiasis in combination with fluconazole (Besterman J., presented at Interscience Conference on Antimicrobial Agents and Chemotherapy IAAC in 2012 in San Francisco, CA, USA). The results of this study indicated that kidney fungal loads in animals receiving both MGCD290 and fluconazole were significantly lower than fungal loads in animals treated with fluconazole alone. These preliminary results on the use of HDAC inhibitors in murine models of candidiasis are conflicting, but there are also major differences between these *in vivo* models. In the future, the relevancy of such *in vivo* experiment will probably need to be attested to provide clear information on the therapeutic potential of the tested compounds. Also, HDAC inhibitors still need to be rigorously tested under a range of experimental conditions to examine both their toxicity and antifungal efficacy. Indeed, as several regulatory and signaling pathways/mechanisms are highly conserved between fungal and human eukaryotic cells, the use of a non-selective HDAC inhibitor (acting upstream these pathways) exposes to the risk of unwanted adverse effects. However, HDAC inhibitors toxicity does not seem to be a major issue yet, but the commercially available HDAC is today were only recently approved. The most common serious adverse events reported with HDAC inhibitors in cancerology were cytopenia (thrombocytopenia, anemia, neutropenia, or leukopenia), pyrexia, infection, sepsis, or cardiac toxicity. Other frequent adverse reactions are fatigue, nausea and diarrhea (Mottamal et al., 2015). In a recent phase 2 clinical study, the combination of oral MGCD290 and fluconazole in patients with moderate or severe vulvo-vaginal candidiasis, although well tolerated, did not significantly improve outcome compared with fluconazole alone (Augenbraun et al., 2013). This result does not support the therapeutic use of this HDAC inhibitor in this particular clinical context. Also, there is currently no available data suggesting that patients treated with HDACs inhibitors are less susceptible to Candida infection, and more specifically designed studies, among onco-hematological patients, are needed to answer this issue. Thus, for now, the clinical utility of HDAC inhibitors remains to be validated, and further research is more necessary than ever. The recent and expanding use of the *Galleria mellonella* larvae model, which *inter alia* allows high-throughput screening of chemical libraries for the discovery of new antifungal compounds will probably facilitate the discovery of more selective and efficient HDAC inhibitors (Lionakis, 2011).

## Conclusion

The activity of HDAC proteins is essential for the functionality of chromatin in all eukaryotic cells. In *Candida* species, most HDACs contribute to life cycle regulation, morphogenic plasticity, and biofilm formation; they are also involved in azole- and echinocandin-resistance. Therefore, their genetic or chemical inhibition can affect yeast virulence and its capacity to form biofilms while also enhancing the efficacy of existing antifungal drugs, even toward resistant strains. Three HDAC inhibitors now have FDA-approval for clinical use and a dozen compounds are included in clinical trials for cancer therapy. Obviously, the development of fungal-specific drugs would have a high clinical impact as they would avoid inhibition of endogenous host HDACs, therefore probably reducing side effects. These molecules already represent a great potential to create new antifungal treatments, especially given their behavior and how well they are tolerated in patients. Along with other compounds targeting innovative machineries (i.e., mitochondrial function, glycosylphosphatidylinositol biosynthesis, vesicle transport) which are currently in preclinical development, they may be incorporated in the antifungal pipeline. However, *in vivo* tests should be expanded to include more diverse animal models, including invertebrates.

## Author Contributions

All authors listed, have made substantial, direct and intellectual contribution to the work, and approved it for publication.

## Conflict of Interest Statement

The authors declare that the research was conducted in the absence of any commercial or financial relationships that could be construed as a potential conflict of interest.
