# Supplementary material for: Histone Deacetylases and Their Inhibition in Candida Species
Source: Front Microbiol. 2016 Aug 5;7:1238. doi: 10.3389/fmicb.2016.01238 (PMC4974301; doi:10.3389/fmicb.2016.01238)
Supplement: Supplementary file 1 [file Table_1.PDF]

Supplementary Table 1

Histone deacetylases and their inhibition in *Candida* species

Cécile Garnaud, Morgane Champleboux, Danièle Maubon, Muriel Cornet*, Jérôme Govin*

*** Correspondence:**
Muriel Cornet
mcornet@chu-grenoble.fr
Jérôme Govin
Jerome.Govin@inserm.fr

Class I

	Ca_Rpd31	Ca_Rpd32	Cg_Rpd3	Sc_Rpd3	Ca_Hos2	Cg_Hos2	Sc_Hos2	Ca_Hos1	Cg_Hos1	Sc_Hos1
Ca_Rpd31	100	74	76	75	47	44	45	30	31	29
Ca_Rpd32	74	100	86	84	49	47	46	30	33	29
Cg_Rpd3	76	86	100	93	50	49	47	29	31	29
Sc_Rpd3	75	84	93	100	51	48	47	29	31	29
Ca_Hos2	47	49	50	51	100	65	66	31	32	32
Cg_Hos2	44	47	49	48	65	100	82	31	33	29
Sc_Hos2	45	46	47	47	66	82	100	33	34	31
Ca_Hos1	30	30	29	29	31	31	33	100	37	37
Cg_Hos1	31	33	31	31	32	33	34	37	100	47
Sc_Hos1	29	29	29	29	32	29	31	37	47	100

Class II

	Ca_Hda1	Cg_Hda1	Sc_Hda1	Ca_Hos3	Cg_Hos3	Sc_Hos3
Ca_Hda1	100	53	54	21	20	20
Cg_Hda1	53	100	77	23	22	22
Sc_Hda1	54	77	100	21	21	20
Ca_Hos3	21	23	21	100	54	52
Cg_Hos3	20	22	21	54	100	76
Sc_Hos3	20	22	20	52	76	100

Class III

	Ca_Hst2	Cg_Hst2	Sc_Hst2	Ca_Sir2	Sc_Sir2	Cg_Sir2	Sc_Hst1	Cg_Hst1	Ca_Hst1	Cg_Hst4	Sc_Hst4	Ca_Hst3	Cg_Hst3	Sc_Hst3
Ca_Hst2	100	48	45	38	34	36	34	33	34	27	27	30	28	29
Cg_Hst2	48	100	50	36	35	33	33	33	31	26	26	25	26	26
Sc_Hst2	45	50	100	35	33	32	32	33	33	25	26	26	26	23
Ca_Sir2	38	36	35	100	36	38	38	36	40	25	24	30	31	29
Sc_Sir2	34	35	33	36	100	62	63	58	42	24	25	27	28	28
Cg_Sir2	36	33	32	38	62	100	66	56	43	22	25	29	28	29
Sc_Hst1	34	33	32	38	63	66	100	56	45	24	26	28	28	28
Cg_Hst1	33	33	33	36	58	56	56	100	43	23	25	29	29	31
Ca_Hst1	34	31	33	40	42	43	45	43	100	27	26	30	29	31
Cg_Hst4	27	26	25	25	24	22	24	23	27	100	56	32	33	35
Sc_Hst4	27	26	26	24	25	25	26	25	26	56	100	33	32	32
Ca_Hst3	30	25	26	30	27	29	28	29	30	32	33	100	43	48
Cg_Hst3	28	26	26	31	28	28	28	29	29	33	32	43	100	58
Sc_Hst3	29	26	23	29	28	29	28	31	31	35	32	48	58	100

Supplementary Table 1. Percent identity matrix of full length HDACs from *C. albicans, C. glabrata* and *S. cerevisiae.*

Full length HDAC proteins from *C. albicans*, *C. glabrata* and *S. cerevisiae* have been aligned with with Clustal Omega (McWilliam et al., 2013), where this information has been downloaded. Each class of HDACs has been treated independently. This information corresponds to the phylogenic trees presented in Fig. 1.

Supplemental references

McWilliam, H., Li, W., Uludag, M., Squizzato, S., Park, Y. M., Buso, N., et al. (2013). Analysis Tool Web Services from the EMBL-EBI. *Nucleic Acids Res.* 41, W597-600. doi:10.1093/nar/gkt376.
