# Supplementary material for: Histone Deacetylases and Their Inhibition in Candida Species
Source: Front Microbiol. 2016 Aug 5;7:1238. doi: 10.3389/fmicb.2016.01238 (PMC4974301; doi:10.3389/fmicb.2016.01238)
Supplement: Supplementary file 2 [file Table_2.PDF]

Supplementary Table 2

Histone deacetylases and their inhibition in *Candida* species

Cécile Garnaud, Morgane Champleboux, Danièle Maubon, Muriel Cornet*, Jérôme Govin*

*** Correspondence:**
Muriel Cornet
mcornet@chu-grenoble.fr
Jérôme Govin
Jerome.Govin@inserm.fr

	Ca_Hst3_HDA	Hs_Sirt1_HDA	Ca_Hst1_HDA	Ca_Sir2_HDA	Ca_Hst2_HDA	Hs_Sirt2_HDA	Hs_Sirt3_HDA	Hs_Sirt6_HDA	Hs_Sirt7_HDA	Hs_Sirt4_HDA	Hs_Sirt5_HDA	Ca_Set3_HDA	Ca_Hos3_HDA	Ca_Hda1_HDA	Hs_Hdac7_HDA	Hs_Hdac9_HDA	Hs_Hdac4_HDA	Hs_Hdac5_HDA	Hs_Hdac6_HDA2	Hs_Hdac10_HDA	Hs_Hdac6_HDA2	Ca_Hos1_HDA	Hs_Hdac8_HDA	Ca_Hos2_HDA	Hs_Hdac3_HDA	Hs_Hdac1_HDA	Hs_Hdac2_HDA	Ca_Rpd31_HDA	Ca_Rpd32_HDA	*C. albicans*		*H. sapiens*		
																														Min	Max	Min	Max	
Ca_Hst3_HDA	100	33	34	32	32	33	30	23	26	23	21	15	14	16	11	15	13	12	14	12	12	14	16	16	15	15	16	14	14	14	100	11	33	Ca_Hst3_HDA
Hs_Sirt1_HDA	33	100	51	46	41	45	42	24	23	28	27	16	12	12	14	14	13	14	15	14	13	12	15	16	15	15	15	17	16	12	51	13	100	Hs_Sirt1_HDA
Ca_Hst1_HDA	34	51	100	55	40	38	39	24	23	28	26	14	11	15	14	16	13	14	14	13	13	15	17	15	13	14	14	14	14	11	100	13	51	Ca_Hst1_HDA
Ca_Sir2_HDA	32	46	55	100	40	41	43	25	27	27	28	13	8	13	15	15	14	12	11	13	11	8	14	12	11	10	10	14	11	8	100	10	46	Ca_Sir2_HDA
Ca_Hst2_HDA	32	41	40	40	100	48	46	26	25	24	23	15	12	14	13	14	14	14	12	11	13	11	13	14	12	15	14	18	16	11	100	11	48	Ca_Hst2_HDA
Hs_Sirt2_HDA	33	45	38	41	48	100	54	27	24	27	24	12	10	17	13	14	13	14	11	11	13	12	13	14	13	14	12	15	14	10	48	11	100	Hs_Sirt2_HDA
Hs_Sirt3_HDA	30	42	39	43	46	54	100	28	27	28	25	10	13	16	15	18	16	15	13	12	14	11	14	12	13	14	15	15	13	11	46	12	100	Hs_Sirt3_HDA
Hs_Sirt6_HDA	23	24	24	25	26	27	28	100	41	28	23	9	11	13	14	15	13	15	10	12	12	12	12	10	10	12	11	12	12	10	26	10	100	Hs_Sirt6_HDA
Hs_Sirt7_HDA	26	23	23	27	25	24	27	41	100	29	25	11	13	10	12	14	13	12	10	10	10	14	9	13	14	14	14	15	14	10	27	9	100	Hs_Sirt7_HDA
Hs_Sirt4_HDA	23	28	28	27	24	27	28	28	29	100	28	11	11	13	12	15	17	15	13	14	13	12	14	16	17	13	13	14	12	11	28	12	100	Hs_Sirt4_HDA
Hs_Sirt5_HDA	21	27	26	28	23	24	25	23	25	28	100	11	13	9	12	12	13	12	12	14	12	13	11	12	12	13	12	12	9	9	28	11	100	Hs_Sirt5_HDA
Ca_Set3_HDA	15	16	14	13	15	12	10	9	11	11	11	100	14	16	15	17	18	14	13	14	13	8	15	13	13	11	11	17	16	8	17	9	18	Ca_Set3_HDA
Ca_Hos3_HDA	14	12	11	8	12	10	13	11	13	11	13	14	100	24	23	23	21	20	25	24	24	17	18	21	20	18	18	16	16	8	100	10	25	Ca_Hos3_HDA
Ca_Hda1_HDA	16	12	15	13	14	17	16	13	10	13	9	16	24	100	41	43	44	42	45	40	42	23	28	30	26	27	28	27	26	13	100	9	45	Ca_Hda1_HDA
Hs_HDAC7_HDA	11	14	14	15	13	13	15	14	12	12	12	15	23	41	100	73	77	72	50	43	43	19	23	24	23	25	24	23	23	11	41	12	100	Hs_HDAC7_HDA
Hs_HDAC9_HDA	15	14	16	15	14	14	18	15	14	15	12	17	23	43	73	100	77	78	46	40	42	21	24	25	24	25	25	24	23	14	43	12	100	Hs_HDAC9_HDA
Hs_HDAC4_HDA	13	13	13	14	14	13	16	13	13	17	13	18	21	44	77	77	100	79	51	41	43	19	23	26	24	26	25	26	26	13	44	13	100	Hs_HDAC4_HDA
Hs_HDAC5_HDA	12	14	14	12	14	14	15	15	12	15	12	14	20	42	72	78	79	100	47	39	42	19	23	26	26	26	26	25	25	12	42	12	100	Hs_HDAC5_HDA
Hs_HDAC6_HDA2	14	15	14	11	12	11	13	10	10	13	12	13	25	45	50	46	51	47	100	54	49	24	27	25	23	26	26	24	24	11	45	10	100	Hs_HDAC6_HDA2
Hs_HDAC10_HDA	12	14	13	13	11	11	12	12	10	14	14	14	24	40	43	40	41	39	54	100	56	20	24	23	22	22	22	22	22	11	40	10	100	Hs_HDAC10_HDA
Hs_HDAC6_HDA2	12	13	13	11	13	13	14	12	10	13	12	13	24	42	43	42	43	42	49	56	100	20	23	26	24	25	25	25	25	11	42	10	100	Hs_HDAC6_HDA2
Ca_Hos1_HDA	14	12	15	8	11	12	11	12	14	12	13	8	17	23	19	21	19	19	24	20	20	100	38	36	32	36	36	33	33	8	100	11	38	Ca_Hos1_HDA
Hs_HDAC8_HDA	16	15	17	14	13	13	14	12	9	14	11	15	18	28	23	24	23	23	27	24	23	38	100	42	43	44	44	41	45	13	45	9	100	Hs_HDAC8_HDA
Ca_Hos2_HDA	16	16	15	12	14	14	12	10	13	16	12	13	21	30	24	25	26	26	25	23	26	36	42	100	63	58	56	55	56	12	100	10	63	Ca_Hos2_HDA
Hs_HDAC3_HDA	15	15	13	11	12	13	13	10	14	17	12	13	20	26	23	24	24	26	23	22	24	32	43	63	100	66	66	60	66	11	66	10	100	Hs_HDAC3_HDA
Hs_HDAC1_HDA	15	15	14	10	15	14	14	12	14	13	13	11	18	27	25	25	26	26	26	22	25	36	44	58	66	100	94	67	70	10	70	12	100	Hs_HDAC1_HDA
Hs_HDAC2_HDA	16	15	14	10	14	12	15	11	14	13	12	11	18	28	24	25	25	26	26	22	25	36	44	56	65	94	100	68	70	10	70	11	100	Hs_HDAC2_HDA
Ca_Rpd31_HDA	14	17	14	14	18	15	15	12	15	14	12	17	16	27	23	24	26	25	24	22	25	33	41	55	60	67	68	100	83	14	100	12	68	Ca_Rpd31_HDA
Ca_Rpd32_HDA	14	16	14	11	16	14	13	12	14	12	9	16	16	26	23	23	26	25	24	22	25	33	45	56	66	70	70	83	100	11	100	9	70	Ca_Rpd32_HDA

Supplementary Table 2. Percent identity matrix of the histone deacetylase domains of HDACs from *C. albicans* and human. Sequences have been downloaded from NCBI and aligned with Clustal Omega (McWilliam et al., 2013) and the percent identity matrix was downloaded. Gray shades highlight the level of identity between entries. Set3 has been highlighted in red as the most divergent HDAC from human ones. Minimal and maximal values of the matrix are indicated for histone deacetylase domains from *C. albicans* and *H. sapiens*. HDA, histone deacetylase domain.

Supplemental references

McWilliam, H., Li, W., Uludag, M., Squizzato, S., Park, Y. M., Buso, N., et al. (2013). Analysis Tool Web Services from the EMBL-EBI. *Nucleic Acids Res.* 41, W597-600. doi:10.1093/nar/gkt376.
