# Supplementary material for: Histone Deacetylases and Their Inhibition in Candida Species
Source: Front Microbiol. 2016 Aug 5;7:1238. doi: 10.3389/fmicb.2016.01238 (PMC4974301; doi:10.3389/fmicb.2016.01238)
Supplement: Supplementary file 3 [file Image_1.PDF]

Supplementary Figure 1

Histone deacetylases and their inhibition in *Candida* species

Cécile Garnaud, Morgane Champleboux, Danièle Maubon, Muriel Cornet*, Jérôme Govin*

*** Correspondence:**
Muriel Cornet
mcornet@chu-grenoble.fr
Jérôme Govin
Jerome.Govin@inserm.fr

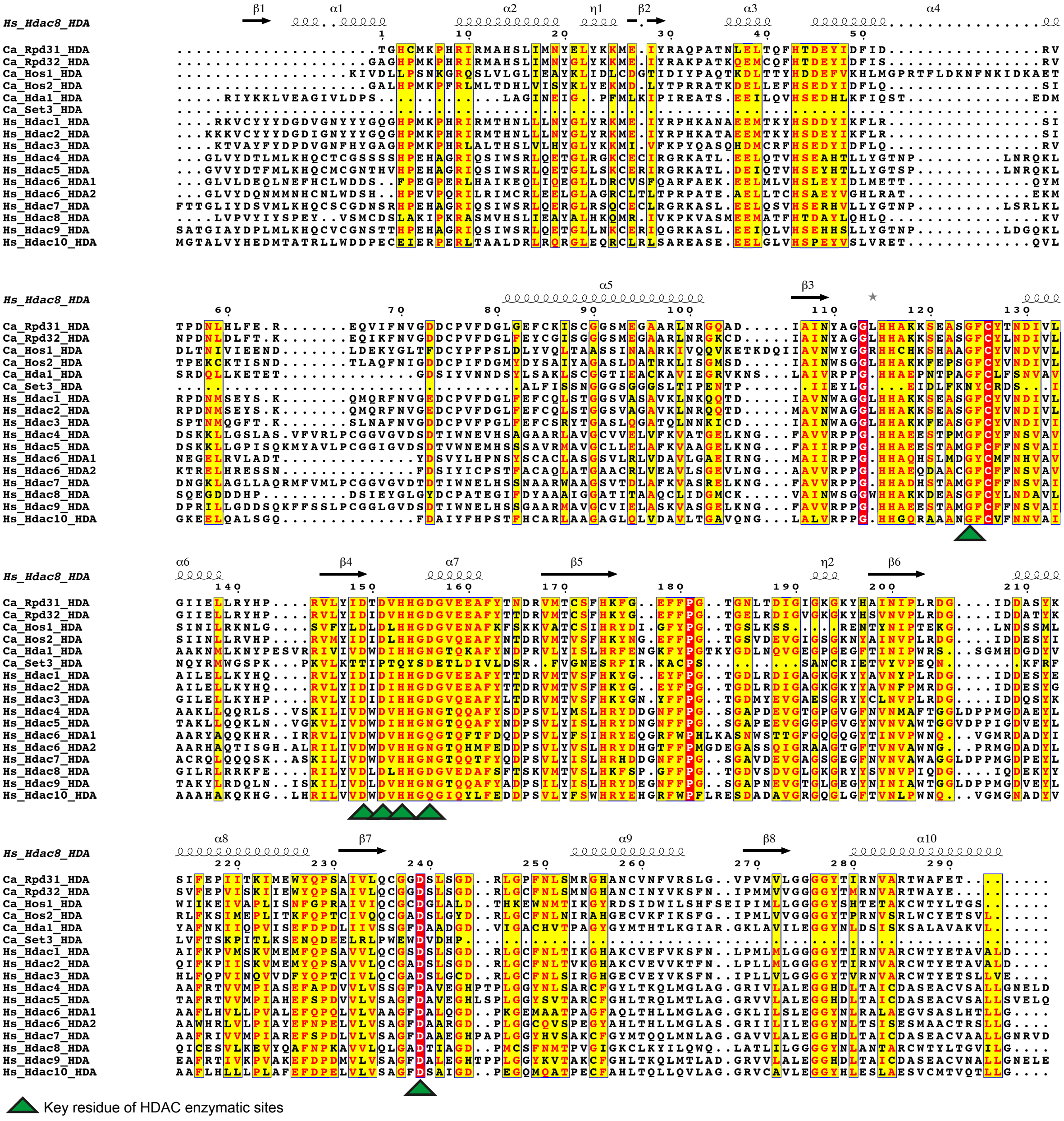

Supplementary Figure 1. Sequence alignment of histone deacetylase domain from human and *C. albicans* HDACs.

Histone deacetylase domains were aligned with NPS@ (Combet et al., 2000) and represented with ESPript 3.0 (Robert and Gouet, 2014). Secondary structure was obtained from HDAC8 (PDB 1T69). Sirtuins were omitted for clarity reasons. Key amino acids of the enzymatic pocket are pointed by a green arrow and obtained from (Lombardi et al., 2011).

Supplemental references

Combet, C., Blanchet, C., Geourjon, C., and Deléage, G. (2000). NPS@: network protein sequence analysis. *Trends Biochem. Sci.* 25, 147–150.

Lombardi, P. M., Cole, K. E., Dowling, D. P., and Christianson, D. W. (2011). Structure, mechanism, and inhibition of histone deacetylases and related metalloenzymes. *Curr. Opin. Struct. Biol.* 21, 735–743. doi:10.1016/j.sbi.2011.08.004.

Robert, X., and Gouet, P. (2014). Deciphering key features in protein structures with the new ENDscript server. *Nucleic Acids Res.* 42, W320-324. doi:10.1093/nar/gku316.
